# Record dry summer in 2015 challenges precipitation projections in Central Europe

**DOI:** 10.1038/srep28334

**Published:** 2016-06-21

**Authors:** René Orth, Jakob Zscheischler, Sonia I. Seneviratne

**Affiliations:** 1 Institute for Atmospheric and Climate Science, ETH Zurich, Universitätstrasse 16, CH-8092 Zurich, Switzerland

## Abstract

Central Europe was characterized by a humid-temperate climate in the 20^th^ century. Climate change projections suggest that climate in this area will shift towards warmer temperatures by the end of the 21^st^ century, while projected precipitation changes are highly uncertain. Here we show that the 2015 summer rainfall was the lowest on record since 1901 in Central Europe, and that climate models that perform best in the three driest years of the historical time period 1901–2015 project stronger drying trends in the 21^st^ century than models that perform best in the remaining years. Analyses of precipitation and derived soil moisture reveal that the 2015 event was drier than both the recent 2003 or 2010 extreme summers in Central Europe. Additionally there are large anomalies in satellite-derived vegetation greenness. In terms of precipitation and temperature anomalies, the 2015 summer in Central Europe is found to lie between historical climate in the region and that characteristic of the Mediterranean area. Even though the models best capturing past droughts are not necessarily generally more reliable in the future, the 2015 drought event illustrates that potential future drying trends have severe implications and could be stronger than commonly assumed from the entire IPCC AR5 model ensemble.

In the recent past Europe experienced extensive heat waves in 2003 (Central Europe)^1^ and 2010 (Eastern Europe)^2^ with severe impacts on economy, public health^3^ and the carbon cycle^4^. These events are in line with a projected increase in the frequency and magnitude of extreme temperatures in Europe in the near future^5^, possibly associated with higher evapotranspiration^6^,^7^. However, there are large uncertainties regarding possible changes in annual precipitation^8^. While extreme temperatures have strong impacts in Europe, drought is associated with some of the largest impacts on agricultural production^9^, ecosystems^10^,^11^, hydrological resources^12^, infrastructure^13^, and thus society. The 2015 Central European summer was comparable with recent extreme events in terms of temperatures (e.g. daily temperature records were broken in various locations), yet here we demonstrate that it was unprecedented in terms of the precipitation deficit. We analyze this event over the Central European domain defined in the IPCC special report on extremes^5^ (henceforth referred to as CEU), and assess it in the context of climate model simulations that contributed to the recent IPCC AR5 report^14^.

## Results

**Figure 1:**
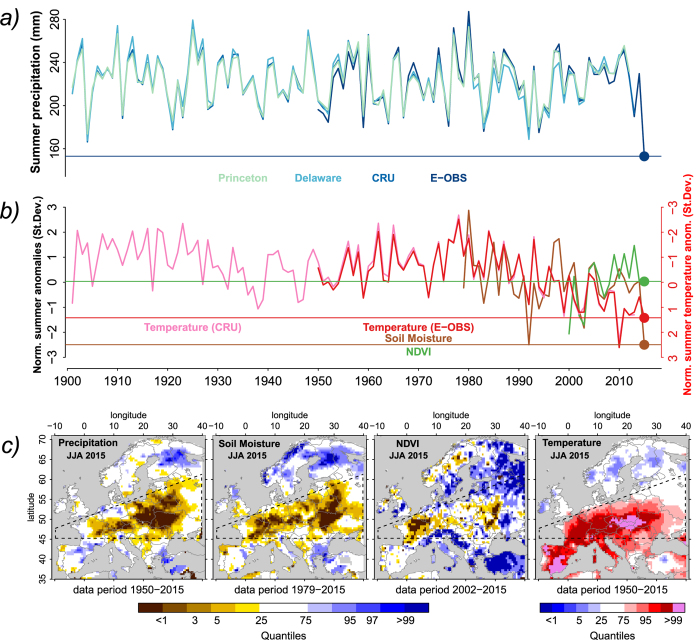
Description of 2015 summer drought. (**a**) Time series of summer (JJA) precipitation averaged across the CEU domain in several observational products. (**b**) Normalized anomalies of temperature, soil moisture and normalized differenced vegetation index (NDVI) averaged across central Europe. Note the inverted temperature axis on the right. (**c**) Mean summer values of all quantities expressed as quantiles across Europe. CEU domain marked with dashed lines. Figure created with R version 3.1.2 (www.R-project.org).

We compare mean summer precipitation over CEU from several products (see Methods) over the historical time period 1901–2015 in Fig. 1a. The summer of 2015 is only available in E-OBS meteorological data^15^. Summer precipitation in 2015 across the CEU domain was substantially lower than in any other year in the past 114 years. This is remarkable, since there is no apparent negative trend over this period (not shown). The quantitative rainfall deficit across CEU in this summer corresponds to 31% of the mean CEU summer precipitation (in the historical time period), and was 46 billion m^3^ larger than the second driest summer in 1904. This amount corresponds to the average yearly water consumption of about 30 million Europeans^16^. Summer temperatures in 2015 across the considered domain were second highest in the considered 115-year record, after 2010 (Fig. 1b). Similar temperature and precipitation anomalies were found in preliminary ERA-Interim reanalysis data^17^ (Figure S1). The extreme rainfall deficit caused the desiccation of soils and consequently strongly impacted vegetation greenness, as evident from reconstructed soil moisture content^18^ and a satellite-based vegetation index^19^ (Methods). The dry and hot weather was most pronounced across Poland, the Czech Republic, Slovakia, western Ukraine and Belarus (Fig. 1c). In June, the temperature anomalies and precipitation deficits were strongest in the southwest of the CEU domain and then moved north-eastwards in July and August (Figure S2). Such a northeast propagation of drought is typical in Europe and might be induced by southerly winds transporting dry air which then tends to incease evaporative demand and temperature further north, and by land-atmosphere interactions^20^. Compared with the 2003 heat wave, the impacted region is shifted towards the northeast. This shift is expected in future climate as an additional warming from stronger land-atmosphere interactions is projected in Central Europe as a response to a drier and warmer summer climate^6^.

We analyze bias-adjusted modelled precipitation (see Methods) from the historical and future time periods in 40 climate models from the Coupled Model Intercomparison Project Phase 5 (CMIP5)^14^ (Table S1). Projected summer precipitation towards the end of the century (2071–2100) depends on the greenhouse gas emission scenario: in a moderate scenario (RCP4.5) the 2015 summer drought would recur with a probability of 5% across all model simulations whereas in the business-as-usual scenario (RCP8.5) this probability increases to 17%. In other words, while the 2015 European summer drought occurred only once in the past 115 years it is expected to become a 1-in-20 year event under the RCP4.5 scenario and a 1-in-6 year event under the RCP8.5 scenario (Methods). For comparison, analyzing all model simulations from the historical time period, we find that the observed 2015 conditions have a return period of more than 300 years. Currently, human emissions are most consistent with the business-as-usual scenario^21^ (RCP8.5), which we will focus on in this study.

**Figure 2:**
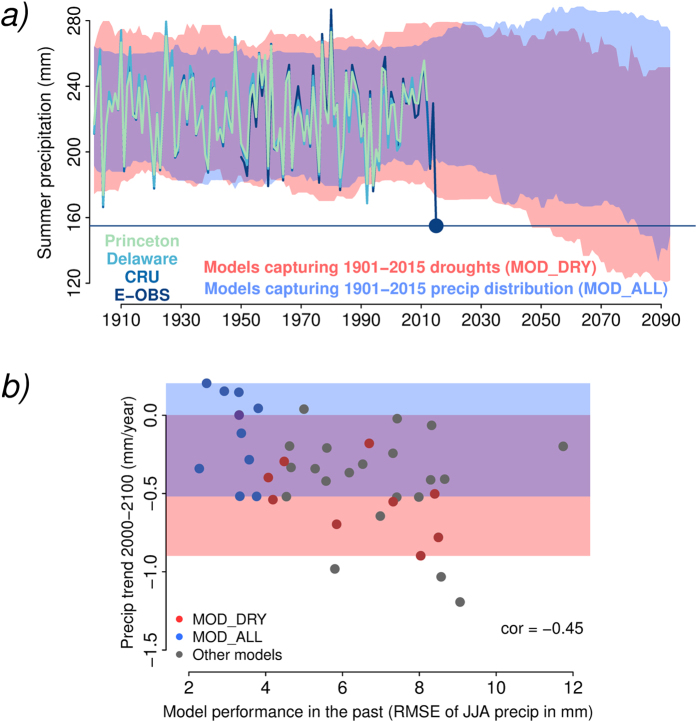
Models capturing best the three most extreme droughts versus models performing best otherwise. (**a**) Time series of observed summer precipitation across the CEU domain along with simulations from the MOD_ALL and MOD_DRY models (see text for details). Results based on simulations using the RCP8.5 scenario. Shading indicates the range between 5th and 95th percentiles, red shading refers to MOD_DRY, blue shading refers to MOD_ALL, and violet shading indicates overlap. (**b**) Precipitation trends during 2000–2100 versus model performance during 1901–2015 (not considering the three driest years) for all CMIP5 models, including the models used in (**a**).

In a next step, we analyze the implications of the 2015 drought for precipitation projections in CEU. To increase the robustness of our analysis we focus on 2015 as well as the second and third driest summers (in 1904 and 1992, respectively). We compare precipitation projections of two subsets from the 40 climate models: (i) the models that perform best for the three driest years during the historical period 1901–2015 (henceforth referred to as MOD_DRY), and (ii) the models that perform best in the remaining 112 years (MOD_ALL). We compute precipitation performance of all models in the respective time frame by comparing observed and modelled distributions of summer precipitation across CEU. To determine the MOD_DRY and MOD_ALL subsets, we calculate the differences (root mean squared error, hereafter referred to as RMSE) between the 3 driest or 112 remaining precipitation values, respectively, of the observed and modeled time series, independently of when they occur (see Methods). We include 10 models in each subset, which are indicated in Table S1. Precipitation projections of these two sets of models are significantly different from 2014 onwards (except for one single year with p = 0.051 which, however, might simply occur by chance; see Methods) as illustrated in Fig. 2a. While the MOD_ALL models project no change in summer precipitation, the MOD_DRY models suggest a substantial future summer drying. Considering all 40 models we find a relationship between projected precipitation trends from 2000–2100 in each model and the respective performance during the historical period excluding the three driest years as assessed with the RMSE (Fig. 2b). The more the models disagree with the historical record across CEU, the stronger is the drying they project. We find similar results with model simulations using the RCP4.5 emission scenario (Figure S3).

The MOD_DRY models benefit from high simulated precipitation variability which, however, seems to degrade their performance over the historical time period as this variability exceeds the observed variability in other years besides the dry extremes. On the other hand the MOD_ALL models might be too strongly adjusted to (non-extreme) observations such that they are not able to capture events such as the 2015 drought. Alternatively it is also possible that this event was an unlikely outlier, which for this reason is not well captured by many models. It is thus not possible to definitely determine whether the MOD_DRY or the MOD_ALL models are more reliable. The fact that the MOD_DRY models project stronger future drying trends, however, raises the question whether the occurrence of the 2015 summer drought renders drier climate projections across the CEU domain more likely. As we focus here on the differences in the simulated precipitation variability across the models we only accounted for their biases in terms of mean precipitation. However, we also investigated output available from 35 CMIP5 models forced according to the RCP8.5 scenario where biases were adjusted more comprehensively (ftp://gdo-dcp.ucllnl.org/pub/dcp/archive/cmip5/global_mon/BCSD/, accessed on 29 March 2016). This dataset was processed using the BCSD methodology (Bias Correction and Spatial Disaggregation, ref. 22), which is based on grid cell-wise quantile mapping. After determining MOD_DRY and MOD_ALL subsets in this dataset, we also find a discrepancy between the respective precipitation projections (Figure S4). For consistency with the previous results, we employ the model data with only adjusted mean biases in the following analyses.

Additionally to the differences in the precipitation projections between the two subsets of models we also find differences in their bias-adjusted temperature projections for the CEU domain (Figure S5). MOD_DRY models suggest more uncertain but overall stronger warming than the MOD_ALL models. In view of the growing body of literature on constraining the CMIP5 model ensemble, these results furthermore suggest that the constraining relationship has to be selected with care as different approaches can lead to contrasting results.

**Figure 3:**
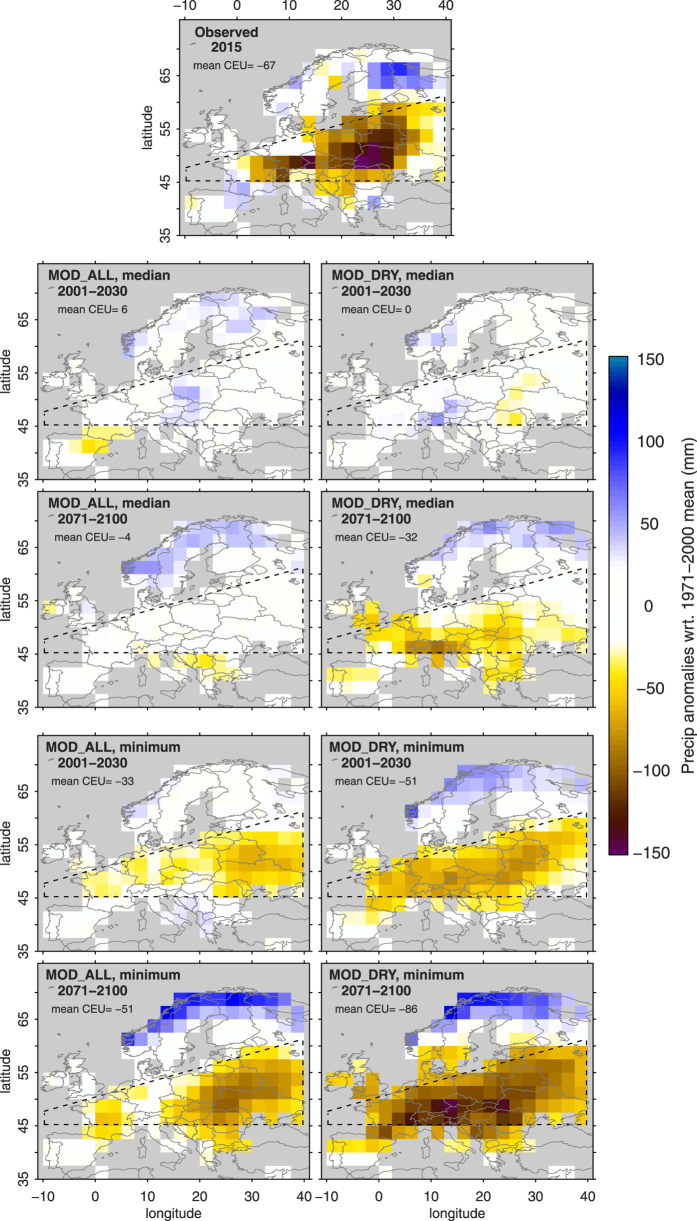
Spatial patterns of CEU median and minimum precipitation. Median and minimum summer precipitation in MOD_ALL and MOD_DRY models. Results shown for 2001–2030 and 2071–2100 time periods (RCP8.5 scenario). Figure created with R version 3.1.2 (www.R-project.org).

We also compare spatial patterns of projected precipitation in the MOD_ALL models and the MOD_DRY models. The precipitation minimum projected by the MOD_ALL models decreases towards the end of the century but does not reach 2015-like conditions (Fig. 3, Methods). In contrast, the MOD_DRY models show a strong drying in both precipitation medians and minima across CEU and the northern Mediterranean. The strongest droughts projected at the end of the century are clearly drier and more widespread than the 2015 event. Interestingly, dry conditions in Central Europe are usually accompanied by wetter-than-average conditions in Northern Europe, both in the 2015 summer’s observations and in model simulations. This is in line with previously reported spatial drought patterns in Europe^23^.

**Figure 4:**
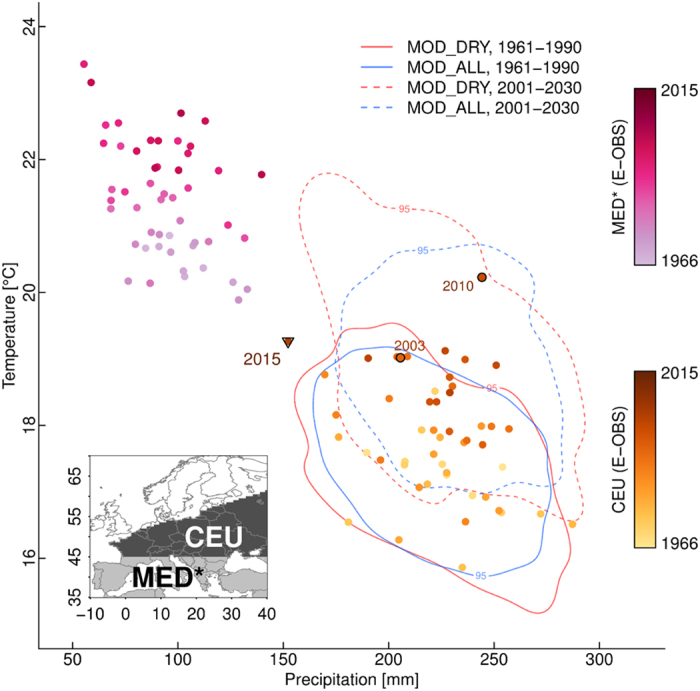
2015 summer climate conditions halfway between CEU and MED* climate. Summer temperature and precipitation in CEU and the Mediterranean region during the last 50 years. Contour lines (95%) show bias-adjusted CMIP5 model results for 1961–1990 and 2001–2030 time periods (RCP8.5 scenario). Note that the Mediterranean region used in this study (MED*, 35–45°N, 10°W–40°E) is slightly smaller than the MED region in ref. 5 (30–45°N, 10°W–40°E), because the E-OBS data coverage is very sparse south of 35°N. Figure created with R version 3.1.2 (www.R-project.org).

Climate model projections suggest that a northward shift of the current Mediterranean climate is to be expected^6^. The constellation of summer precipitation and temperature in 2015 cannot be attributed unambiguously to the historical climate of CEU. In the temperature-precipitation space the 2015 event is located in almost equal distance to the driest/warmest years previous to 2015 in CEU, and the wettest/coldest years in the Mediterranean (Fig. 4). Near-term projections from both the MOD_ALL and the MOD_DRY models indicate a warming in CEU, however, the latter models project a stronger warming which is furthermore accompanied by a drying, consistent with the deviation of summer 2015 from the recent decades. Similar results are obtained with the more moderate emission scenario (Figure S6). Accordingly, if the 2015 rainfall deficit were to recur in the future as projected by the MOD_DRY models under the high emission scenario, it would likely be accompanied by much hotter temperatures, rendering the expected impacts more severe.

We have shown that the 2015 summer was a concurrent dry and hot extreme in large parts of Central Europe, and that models that best capture the three strongest past droughts in that region (MOD_DRY) suggest that such events might be more likely in the future than commonly assumed. Compound extremes can cause profound impacts across various sectors^24^,^25^,^26^, particularly because human and ecological systems in Central Europe are less adapted to such conditions as for instance in the Mediterranean. Among others^12^, the following impacts could be of particular high relevance in Central Europe: (1) Low soil moisture challenges agriculture and reduces yields of summer crops^27^ such as maize, potatoes, or cereals; (2) Low river levels impede navigation and threaten fish^12^; (3) droughts can cause shrinking and swelling of soils leading to building damage^13^; (4) livestock farming and forestry face problems in very dry conditions^28^; and (5) increased hot extremes from drought feedbacks^29^,^30^ can cause increased mortality especially amongst elderly people^3^. These combined consequences underline the importance of an improved understanding of (concurrent) droughts and heat waves in future climate in order to foster adaptation to extreme events as well as the mitigation of climate change.

## Methods

### Observation-based datasets

We employ precipitation data from several products: (i) Delaware dataset^31^, (ii) Princeton forcing data^32^, (iii) CRU dataset^33^, and (iv) E-OBS gridded observations^15^. Additionally we use temperature data from the CRU and E-OBS datasets. To assess the state of the vegetation we employ normalized difference vegetation index^19^ (NDVI) data. We use the Terra Moderate resolution Imaging Spectra-radiometer (MODIS) Collection 6 standard NDVI product (WWW-MOD13C1, available at https://lpdaac.usgs.gov/dataset_discovery/modis/modis_products_table/mod13c1_v006) covering the time period 2000–2015. We aggregated the data through spatial averaging to 0.5° × 0.5° spatial resolution and monthly temporal resolution. All selected data are based on MODIS land surface reflectance data and are thoroughly corrected for atmospheric disturbances. Furthermore we apply a strict quality control to minimize non-vegetative signals (i.e., snow, cloud and cirrus). We use a simple water balance model^34^ forced with E-OBS temperature and precipitation to estimate soil moisture (as in ref. 18). Furthermore net radiation data is needed to force this model. Instead of using satellite-derived data as in ref. 18, we employ here net radiation from ERA-Interim as this is timely available to compute soil moisture of the summer 2015. This data allows us to furthermore extend the soil moisture reconstruction into the past until 1979.

Observational data displayed in Figs 1b and S1b is normalized through subtracting the respective mean and dividing by the respective standard deviation. Therefore, we use the reference time period 1981–2010 for temperature and soil moisture, and 2002–2015 for NDVI.

### Processing of CMIP5 data

We adjust the precipitation bias of each CMIP5 model and each observational product against a reference. As an observational reference we employ the mean of the summer precipitation estimates of the Delaware, Princeton and CRU datasets. The biases are determined over the time period 1950–2012. Bias-adjustment is applied with respect to the mean precipitation across the CEU domain. The same bias adjustment is applied to the entire time series of each product, i.e. the bias is assumed to be stationary. This procedure ensures to adjust model biases but also the known precipitation undercatch in the E-OBS gridded observations^35^. In Figs 4 and S5 also temperature biases of the CMIP5 models are adjusted against E-OBS summer temperature mean during 1950–2012.

Probability of occurrence of a summer that is at least as dry as 2015 is determined over the time periods 1901–2015 and 2071–2100 using data from all available CMIP5 models (Table S1). Similar results are found when first computing the probability for each model individually and then averaging across all models.

As a basis for the determination of the MOD_DRY and MOD_ALL model subsets we assess the performance of modelled summer precipitation averaged over CEU by comparing the observed and modeled precipitation distribution. For this purpose we sort the 1901–2015 modelled and observed precipitation amounts, respectively, and calculate the root mean squared deviation between the sorted values. In the case of MOD_DRY only the three driest years are considered, in the case of MOD_ALL all other 112 years are considered.

The 5th-to-95th percentile range in Fig. 2a is computed for each year. We use data from MOD_DRY and MOD_ALL, respectively. Additionally to the analyzed year we consider the 7 previous years and the 7 subsequent years (assuming no trend across these years). Hence percentile ranges of a given year are computed from 9×15 = 135 precipitation estimates. The significance of the difference of the summer precipitation between these 2 model ensembles is assessed with a Welch’s t-test using the 135 estimates of each ensemble in each year. Normality of the respective distributions is confirmed with a Shapiro-Wilk test (at the 5%-level).

Precipitation medians and minima over the two 30-year time periods used in Fig. 3 are computed with the following procedure to ensure the spatial coherence of the derived patterns. From each considered model we select the year closest to the median and the minimum of precipitation across the CEU domain, respectively. Using these selected years we then compute the mean precipitation anomaly map across a subset of models.

All data analysis and figures were done using the R programming language^36^ version 3.1.2.

## Additional Information

**How to cite this article**: Orth, R. *et al.* Record dry summer in 2015 challenges precipitation projections in Central Europe. *Sci. Rep.* **6**, 28334; doi: 10.1038/srep28334 (2016).

## Supplementary Material

Supplementary Information
