# Supplementary material for: Record dry summer in 2015 challenges precipitation projections in Central Europe
Source: Sci Rep. 2016 Jun 21;6:28334. doi: 10.1038/srep28334 (PMC4914956; doi:10.1038/srep28334)
Supplement: Supplementary Information [file srep28334-s1.pdf]

Record dry summer in 2015 challenges precipitation projections in Central Europe

René Orth[1], Jakob Zscheischler[1], and Sonia I. Seneviratne[1]

[1] Institute for Atmospheric and Climate Science, ETH Zurich, Universitätstrasse 16, CH-8092 Zurich, Switzerland (rene.orth@env.ethz.ch)

Supplementary Table 1: List of considered CMIP5 models in this study. We analyzed daily data of precipitation and 2m-temperature from simulations for the historical period (1901-2005), the RCP4.5 scenario (except for models marked with *) and the RCP8.5 scenario. Superscripts MOD_DRY and MOD_ALL indicate which models belong to these groups.

Employed CMIP5 models		
ACCESS1-0 [MOD_DRY]	CSIRO-Mk3-6-0	HadGEM2-ES [MOD_DRY]
ACCESS1-3	EC-EARTH *	inmcm4
bcc-csm1-1-m [MOD_DRY]	FGOALS-g2	IPSL-CM5A-LR
bcc-csm1-1	FIO-ESM [MOD_DRY]	IPSL-CM5A-MR [MOD_DRY]
BNU-ESM	GFDL-CM3	IPSL-CM5B-LR [MOD_ALL]
CanESM2 [MOD_DRY]	GFDL-ESM2G	MIROC5 [MOD_DRY]
CCSM4	GFDL-ESM2M	MIROC-ESM-CHEM [MOD_ALL]
CESM1-BGC	GISS-E2-H-CC	MIROC-ESM [MOD_ALL]
CESM1-CAM5 [MOD_ALL]	GISS-E2-H	MPI-ESM-LR [MOD_ALL]
CMCC-CESM *	GISS-E2-R-CC [MOD_DRY]	MPI-ESM-MR
CMCC-CM [MOD_DRY]	GISS-E2-R	MRI-CGCM3 [MOD_ALL]
CMCC-CMS [MOD_ALL]	HadGEM2-AO	MRI-ESM1 [MOD_ALL] *
CNRM-CM5 [MOD_DRY, MOD_ALL]	HadGEM2-CC	NorESM1-ME [MOD_ALL]
		NorESM1-M

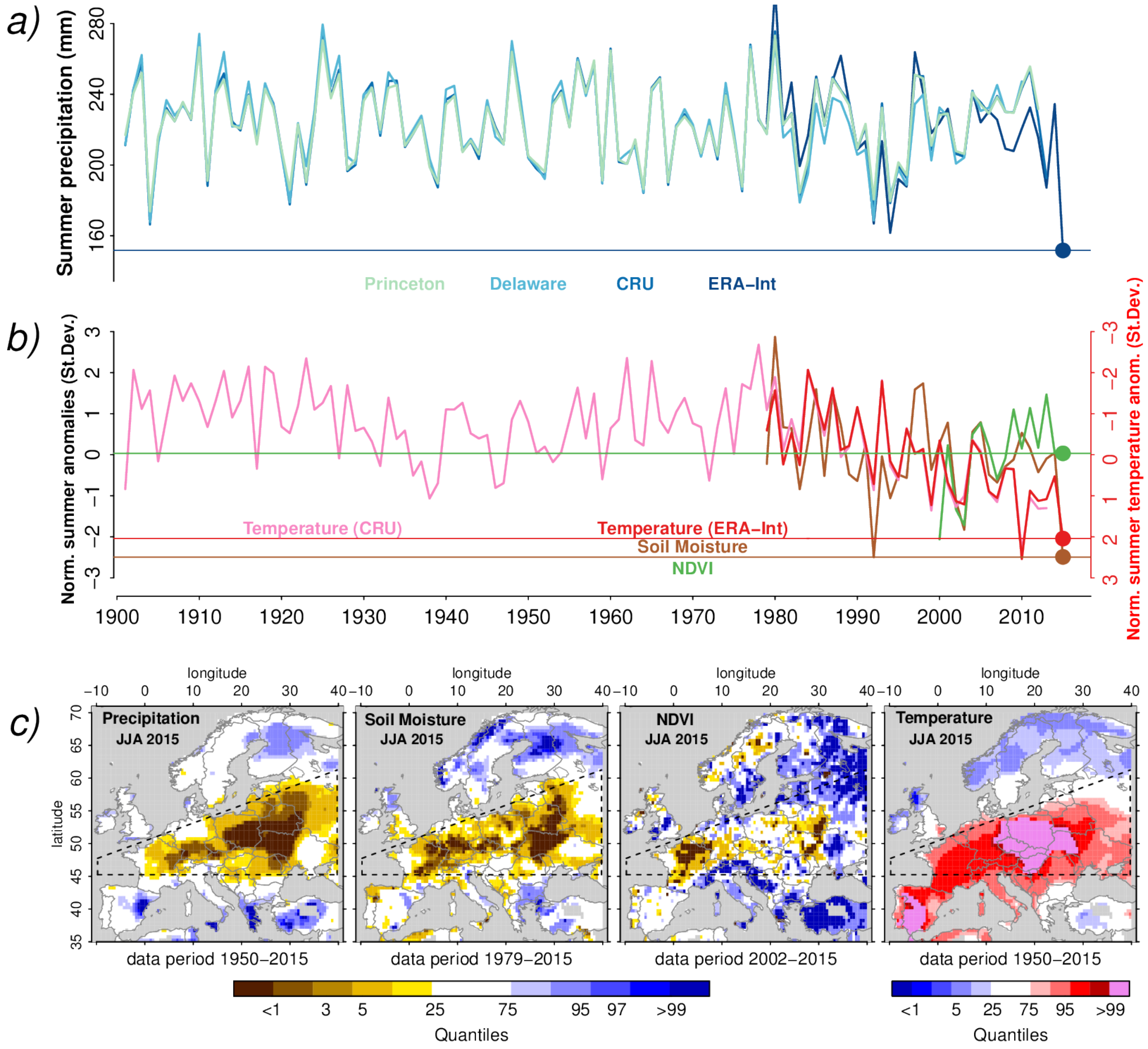

Supplementary Figure 1: Description of the 2015 summer drought with alternative data. Same as in Figure 1 but with ERA-Interim temperature and precipitation data. The soil moisture product is based on E-OBS temperature and precipitation, and on ERA-Interim radiation data. Figure created with R version 3.1.2 (www.R-project.org).

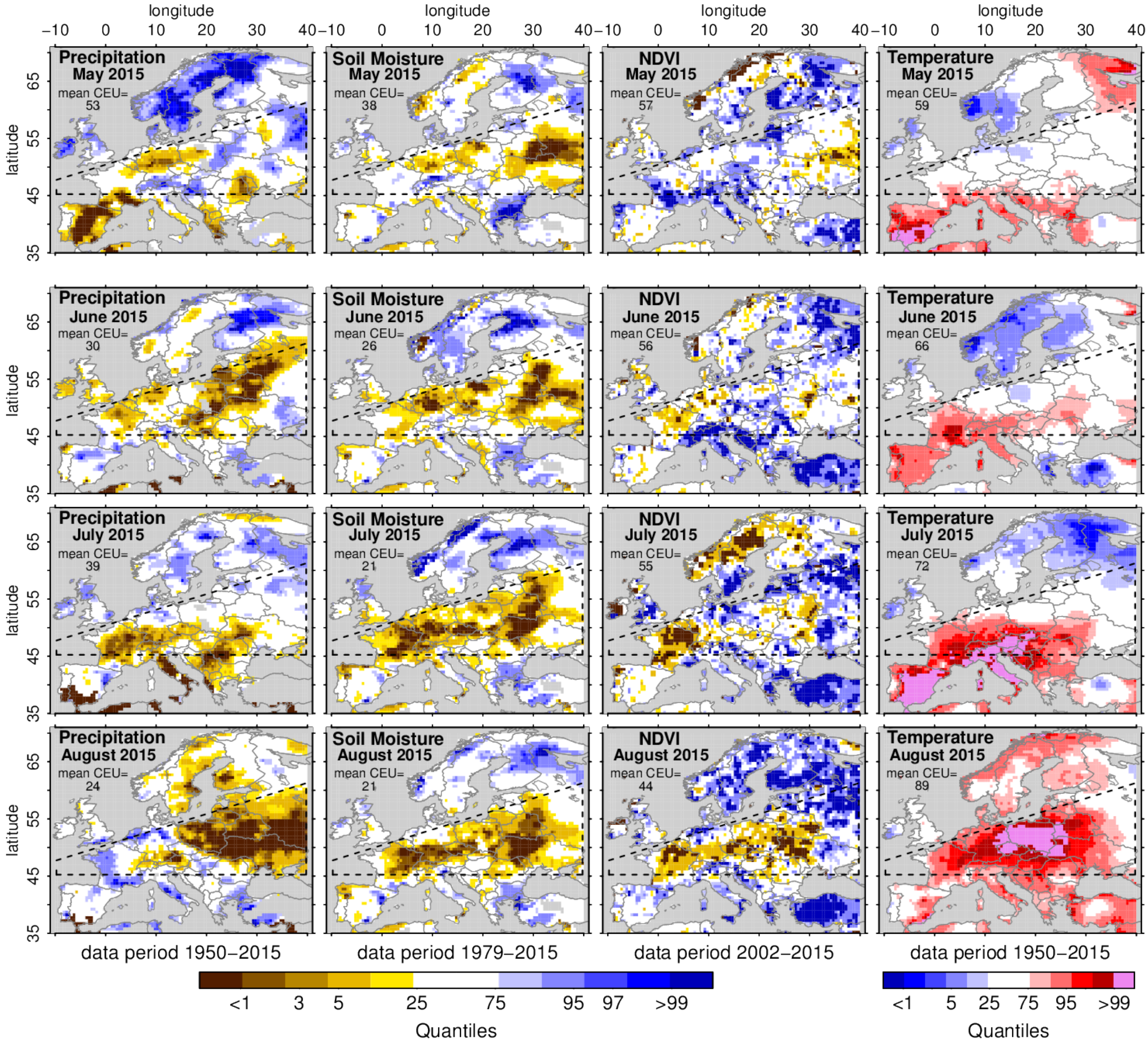

Supplementary Figure 2: Evolution of the 2015 summer drought. Evolution of anomalies of precipitation (E-OBS), soil moisture, NDVI and temperature (E-OBS) during May-August 2015. Figure created with R version 3.1.2 (www.R-project.org).

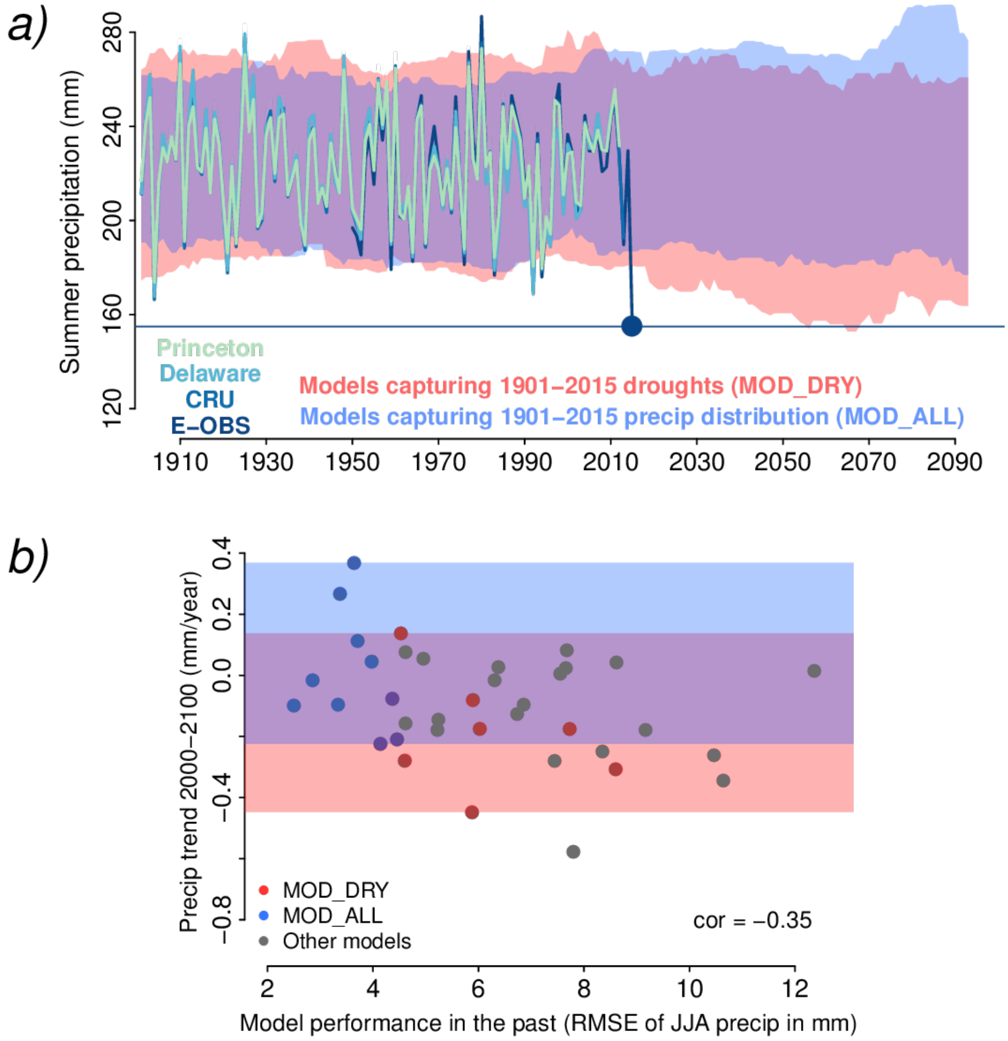

Supplementary Figure 3: Models that best capture droughts versus models performing best otherwise. Same as in Figure 2 but for the RCP4.5 emission scenario.

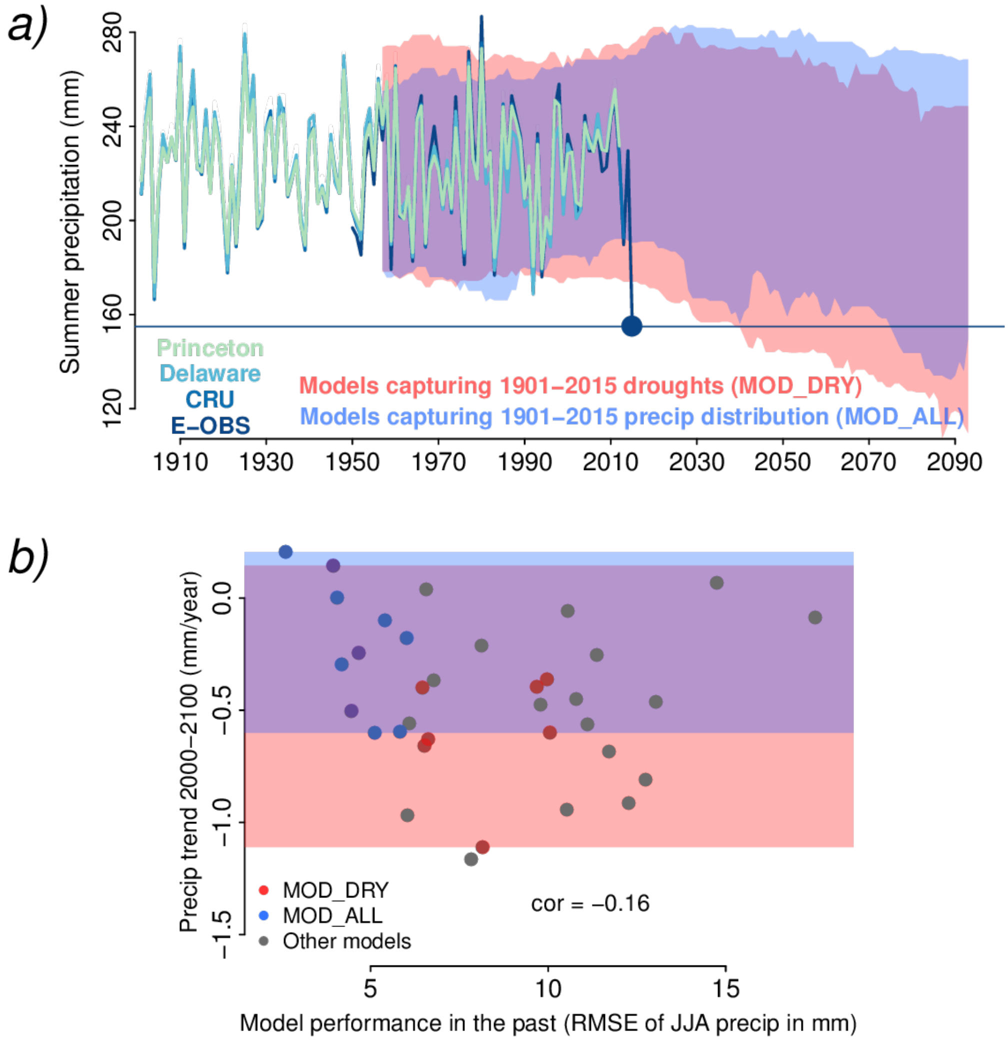

Supplementary Figure 4: Models that best capture droughts versus models performing best otherwise. Same as in Figure 2 but for model data bias-adjusted with the BCSD methodology.

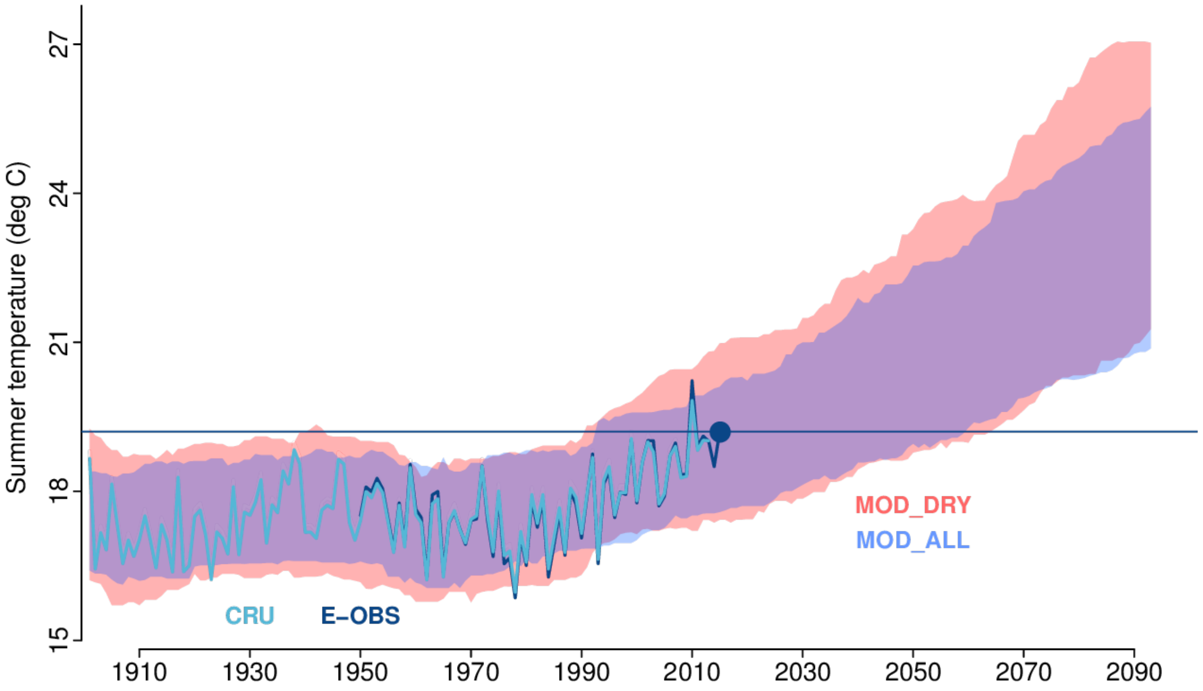

Supplementary Figure 5: Temperature projections, MOD_DRY models vs. MOD_ALL models. Same as in Figure 2a but for mean summer temperature. Results based on the RCP8.5 scenario.

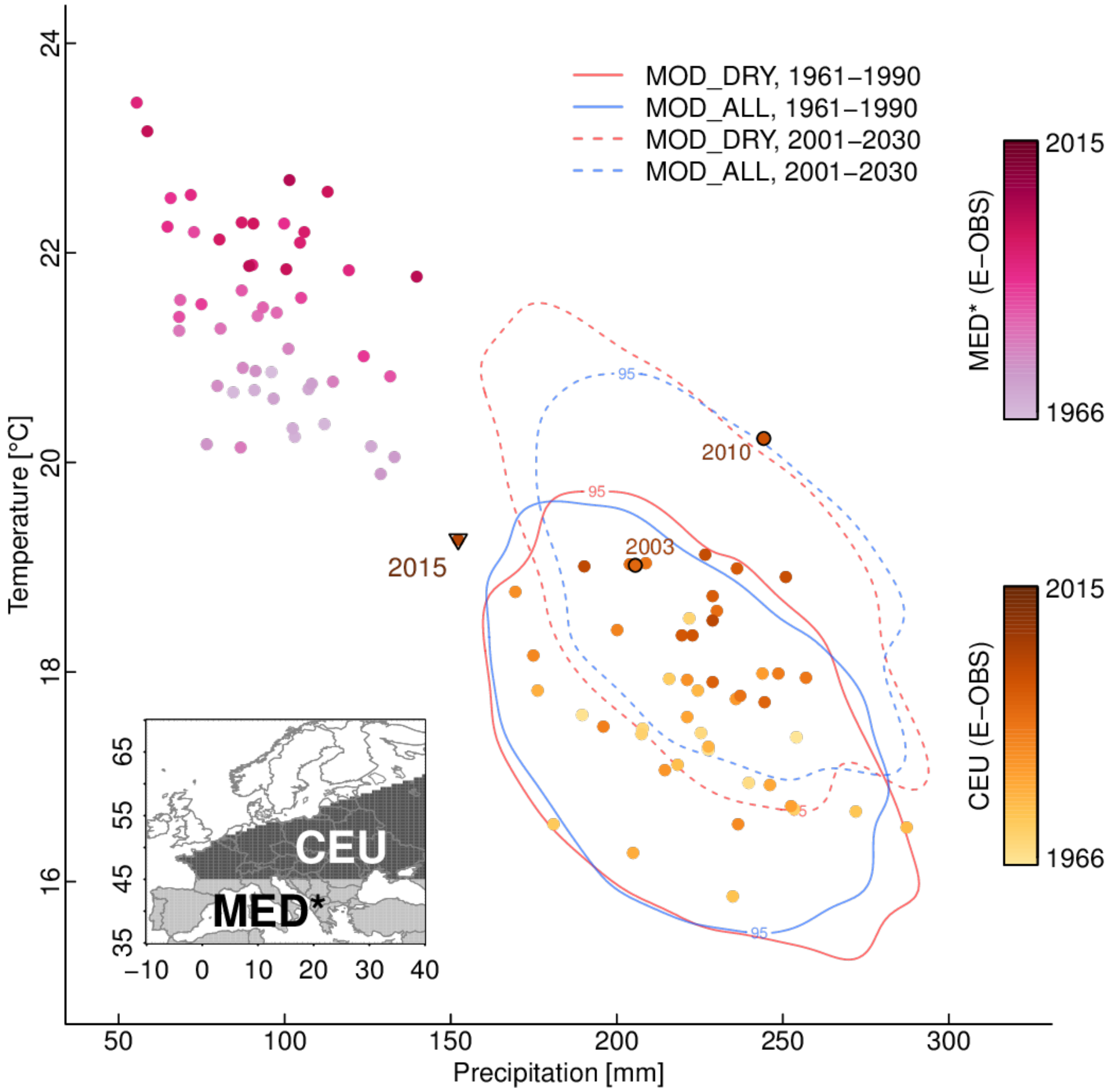

Supplementary Figure 6: 2015 summer climate conditions halfway between CEU and MED* climate. Same as in Figure 4 but for the RCP4.5 emission scenario. Figure created with R version 3.1.2 (www.R-project.org).
